# 77099 Implementation of the Capute Scales and Prechtl’s General Movement Assessment in Infants with Single Ventricle Physiology

**DOI:** 10.1017/cts.2021.737

**Published:** 2021-03-30

**Authors:** TL Johnson, H Raiees-Dana, A Escapita

**Affiliations:** 1 University of Arkansas for Medical Sciences; 2 Arkansas Children’s Hospital

## Abstract

ABSTRACT IMPACT: Through this research, I will transform the standard of care for infants with single ventricle physiology by incorporating the Capute Scales and General Movement Assessment into day-to-day clinical care for these infants, leading to early detection of neurodevelopmental disabilities and access to proven therapies. OBJECTIVES/GOALS: Our objective was to establish a new protocol to detect and quantify developmental delays in multiple domains in infants with single ventricle physiology, a type of congenital heart disease. This population is at high risk for neurodevelopmental disabilities. METHODS/STUDY POPULATION: We implemented a novel protocol using the Capute Scales and General Movement Assessment to evaluate early language, cognitive, and motor development in infants with single ventricle physiology. The infants were evaluated between 1-5 months of age in the cardiac neurodevelopmental program. We defined our primary outcomes as (1) language and (2) cognitive developmental quotients as per the Capute Scales and (3) results of the General Movement Assessment. We hypothesized that infants with single ventricle physiology would have typical language and cognitive development and normal General Movement Assessment results at their initial evaluation. RESULTS/ANTICIPATED RESULTS: We recruited ten infants with single ventricle physiology. All ten infants had typical language development, and nine of the ten had typical cognitive development, as measured by the Capute Scales. All of the infants had gross motor delay. Due to medical instability, we only evaluated four infants with the General Movement Assessment. All four of the infants had a normal result, suggesting that their central nervous system motor pathways were maturing appropriately. In future studies, we will track the neurodevelopmental outcomes of each participant as they mature. We expect to see a decrease in expressive language development and preserved receptive language and cognitive development. DISCUSSION/SIGNIFICANCE OF FINDINGS: The combination of General Movement Assessment and Capute Scales in the evaluation of infants with single ventricle physiology will provide early identification and intervention for these high-risk children, allowing access to proven treatments and therapies.

